# Supporting children’s wellbeing through outdoor time: opportunities to integrate consistent outdoor time into the elementary school day

**DOI:** 10.3389/fpubh.2024.1483862

**Published:** 2024-12-04

**Authors:** Amie K. Patchen, Amanda Edwards, Donald A. Rakow, Genevive R. Meredith

**Affiliations:** ^1^Department of Public and Ecosystem Health, Cornell University, Ithaca, NY, United States; ^2^Public Health Program, Cornell University, Ithaca, NY, United States; ^3^Section of Horticulture, School of Integrative Plant Science, Cornell University, Ithaca, NY, United States

**Keywords:** mental wellbeing, children, elementary schools, nature, outdoor learning, schoolyard

## Abstract

**Background:**

Time in nature supports cognitive, psychological, academic, and health benefits. Outdoor time during school can provide young people with equitable access to these benefits, however, there are within-school constraints. Understanding if and how teachers can frequently and consistently incorporate outdoor time in the schoolyard into their classroom activities can inform broader implementation.

**Methods:**

A mixed-methods observational study was conducted with 17 teachers from five elementary schools. Teachers reported on how they incorporated outdoor time into their classes over an eight-week period. Data on outdoor time, activities, locations, barriers, motivations, and facilitators were collected through surveys on 15 randomly pre-selected days, and through a post-survey.

**Results:**

On the 15 randomly selected days surveyed, classes went outside 78% of the time, ranging from 6–15 outdoor days per class, and 0–285 min per day. Time, activities, locations, motivations, barriers, and facilitators varied across classes and days. The most common activities were free play (*n* = 138) and intentional engagement with nature (*n* = 57). Health benefits (*n* = 68) and having time (*n* = 58) were the most common motivations teachers reported for incorporating outdoor time.

**Implications for school health, policy, practice, and equity:**

Consistent outdoor time in the school day can support students’ wellbeing and academic success. Encouraging free play, highlighting the health benefits, using a systems approach, and flexible implementation may help teachers increase their use of outdoor time during the school day.

**Conclusion:**

Teachers consistently incorporated outdoor time, but implementation varied across classes and days. Implementation findings from this district could help inform practices at other schools.

## Introduction

Outdoor time during the school day has been identified as a promising practice to support academic, health, and wellbeing outcomes (1–6). Time spent outdoors and in nature during childhood brings cognitive, psychological, academic, and health benefits to children, and supports environmental stewardship. Studies have documented positive outcomes including reducing stress (7–9) and increasing attention (10), resilience (11), physical health (12), academic test scores (13), content learning (14, 15), and self-regulation (4), even from short interactions with nature (16). Additionally, guided interactions with nature have been found to support development of environmental attitudes and behaviors (17–19), an important factor at a time when environmental changes are having a substantial impact on human health (20). Overall, however, young people are spending less time outdoors than in previous generations (21, 22), and access to outdoor spaces is not equitable for all children due to environmental and structural barriers (23–25). Further, a lack of engagement with nature compounds pressing problems: young people are experiencing high rates of stress and anxiety (26, 27), and inequitable gaps in academic achievement persist (28).

Schools hold great potential as venues for increasing nature contact for students in ways that open access to all students, particularly as schools work to support students’ mental wellbeing (27) and help students bridge the academic gaps (29). Several recent reviews have found generally positive impacts on students’ mental and physical health, cognitive development, and academic outcomes from school-related outdoor learning across settings (30), holding weekly or biweekly content classes outdoors in various settings (31), and immersive outdoor education experiences (32). Research focused on outdoor learning in the schoolyard has identified benefits related to content learning, student interest or motivation to learn, community involvement, social development, sense of belonging, stress reduction, increased attention, and physical health (1–3, 6). A recent review of research on how nature affects children’s health identified green schoolyards as a major category of nature exposure tied to physical health, mental health, and academic outcomes, though they note that studies did not routinely measure actual use of the schoolyard space (5). In some places nature experiences have been well integrated into the routine function of schools, notably in Scandinavian countries (33). National surveys in Denmark and Norway found that 18% (34) and 69% (35), respectively, of schools practiced “udeskole” (outdoor learning) weekly or biweekly, with a higher prevalence in lower grades in both countries. Rates of outdoor learning and nature exposure during school are not consistent across countries: in a 2018 survey of teachers from 713 schools in the United States (US), United Kingdom (UK), Canada, and Australia that participated in an outdoor learning project, teachers from the US reported significantly less outdoor playtime during the school day for all grades than other countries, and 29% of teachers reported taking their students outside for learning less than once a month (36).

In this project we were particularly interested in the potential of schools to support student wellbeing through integration of outdoor time in the elementary school day at public schools in the US, where many (though not all, particularly in urban locations) have greenspace in the schoolyard, but routine integration of outdoor learning is less common than in other countries (36). We posited that understanding if and how teachers are able to use the greenspace in the schoolyard could support efforts to provide consistent access to nature-rich spaces. Current literature related to three factors—time dose, activities and locations, and barriers guided our project.

### Time dose

Research into the “dose” of nature contact needed to achieve health and cognitive benefits includes many different measures of nature exposure, ranging from residential greenness to specific time exposure to immersive outdoor learning (4). This reflects different approaches taken by different fields, and multiple theories on the mechanisms through which nature exposure affects humans (4). In this project we were interested in the potential benefits from short exposure time—the type of nature exposure that seemed most likely to be consistently integrated into the school day at public elementary schools. Research on time-based exposure for adults indicates that 10–50 min per session can produce mental health benefits (16), and between 120 and 300 min a week in nature is correlated with better health (37). For children, a meta-analysis (4) exploring self-regulation (cognitive, affective, behavioral) benefits from nature exposure included only a handful of studies that directly examined the impact of a short time dose; the studies found benefits after 3 min watching a nature video (38), 10 min in the school garden (39), or 20 min walking in natural parks (40, 41). These studies suggest that short exposure to nature of the type that may be the most likely to be consistently integrated into the elementary school day could provide cognitive and health benefits across the week.

There are, however, limited data indicating the dose of nature students may receive through school activities in the schoolyard. Studies that include reports of the time spent outside during the school day frequently do so based on the time scheduled in the design of a particular program (rather than implementation of outdoor time) or on estimates across a school or time period (10, 42–44). For example, studies have reported that classes participated in ten 40-min outdoor lessons for a specific project (10) and a majority of schools have 15–30 min of scheduled daily recess and estimated spending 66–88% of that recess outdoors annually (42). In other examples, schools estimated missing 3–40 days of outdoor recess annually due to outdoor temperature (43) and teachers were able to utilize an outdoor classroom on 82% of intended days, but did not report the amount of time spent outside on those days (44). These studies provide helpful partial estimates of time students spent outdoors during school hours, but they do not indicate the dose of outdoor time that can be consistently integrated into the school day.

### Activities and locations

School-based nature engagement in the schoolyard can take many forms including instruction focused on nature or sustainability topics (3), outdoor instruction on other topics (e.g., literacy) (45), gardening (2), physical activity (46), and free play (47). Outdoor spaces vary by school but can include playgrounds, blacktop, grass playing fields (46), gardens (2), woods or nature trails, or outdoor classrooms (44). While there is extensive research into the structure and developmental outcomes for specific outdoor learning projects (1–3), and the impact of various schoolyard locations on physical activity (46), there is less documentation of the ways in which individual teachers combine spaces and activities to independently integrate outdoor time into their classes. Structured outdoor learning projects can be an excellent way to engage students with nature, but individual projects have limitations as a means for making outdoor time a consistent part of the school day across the academic year. For instance, projects focused on a particular science topic (3) or activity such as gardening (2) may end when the unit or gardening season ends; costs (6, 48, 49) or the need for buy-in from administrators (50–52) may limit opportunities to engage in specific projects; projects that are not aligned to required curricular topics may not be used (51, 53, 54); and outdoor activities that do align to the curriculum may not be implemented due to instructional (e.g., materials, technology, writing surfaces) or logistical (schedules, staffing, weather, available spaces) considerations (48, 55).

### Barriers

While there are many excellent examples of projects related to environmental education, green schoolyards, outdoor classrooms, and other outdoor learning (1, 56), incorporation of time in nature depends on individual teacher characteristics and school/environmental structures (50, 52, 57). This is because schools are dynamic institutions and teachers encounter many shifting factors that may limit outdoor opportunities (48). Barriers that appear to limit outdoor opportunities include school schedules, resources, appropriate spaces, teacher knowledge, curriculum requirements, staff ratios, safety and liability concerns, policies, support from administrators, weather and clothing, logistical issues, and equity concerns (1, 6, 48–53, 58, 59).

### Study focus

In this project we sought to explore strategies to help teachers overcome barriers to outdoor time, and to increase integration of frequent (e.g., multiple days a week) and consistent (i.e., maintained throughout the academic year) outdoor time into the elementary school day, as a possible means to support student wellbeing. As a step towards integrating outdoor time frequently and consistently across the school year, we wanted to understand the ways in which elementary teachers were able to integrate outdoor time into their class schedule independently (i.e., without the structure of a specific curriculum or outdoor learning project); how long they were able to spend outside; and what facilitated or limited opportunities. Understanding if and how teachers are able to consistently incorporate ‘useful doses’ of outdoor time into their classes could help increase uptake of this promising practice. Thus, this action-research project focused on these specific research questions:

1. To what extent can elementary teachers consistently incorporate outdoor time during the school day? (minutes, days)
2. In what ways were elementary teachers able to consistently incorporate outdoor time? (location, activity)
3. What motivated teachers to consistently incorporate outdoor time? (reasons)
4. What helped teachers consistently incorporate outdoor time? (facilitators)
5. What limited teachers’ opportunities to consistently incorporate outdoor time? (barriers)

To examine this, we asked teachers to try to take their classes outdoors at times other than daily recess for 8 weeks and report to us on what they were able to accomplish, what supports helped increase outdoor time, what motivated them, and what limited opportunities.

## Materials and methods

This study was part of an action research project that prioritized implementation of outdoor time in elementary schools by providing guidance, support, and resources to motivated teachers, those who wanted to increase outdoor time for their classes. This study was reviewed and approved by the Cornell University Institutional Review Board (Cornell IRB # 1812008455). Using a Design-Based Implementation Research (DBIR) approach (60) we worked with teachers to understand their current use of outdoor time on the school grounds, and facilitators and barriers to incorporating outdoor time into their classes. This resulted in the collaborative development of a guide with teacher-elucidated strategies (61). Phase 1 of the study (reported elsewhere) examined barriers that limited teachers’ access to outdoor time (48). In this phase (Phase 2, reported here), we examine teachers’ opportunities to independently integrate outdoor time into their school day when given encouragement, a flexible structure, and modest tangible supports. Teachers participated in a workshop and agreed to try to incorporate outdoor time in addition to daily school recess as a frequent, consistent, part of their class time for a period of 8 weeks. Data collection occurred over the 8 weeks (15 randomly selected school days), and at one time-point after the project ended.

### Participants

Participants included 17 teachers from five elementary schools (grades preK-5) in one school district in the northeastern US. The school district is in a small city located in a predominantly rural area. Three schools had extensive greenspace on the school grounds including fields, nature trails, and outdoor classrooms consisting of log seats. Two schools were in the city center and had cultivated greenspace (garden boxes, pollinator garden) on the school grounds, as well as walkable access to nearby city parks that teachers occasionally used. Participants were recruited through short presentations at virtual school staff meetings and digital flyers about the project. Participants were “early adopters” and volunteered for the project because they were interested in increasing the time their classes spent outdoors. Recruited teachers included four pre-K, four kindergarten, one 1^st^ grade, two 2^nd^ grade, two 3^rd^ grade, three 5^th^ grade, and one who worked across K-5 (Table 1). One teacher, “P,” was removed from the sample as survey responses were consistently not related to the questions asked.

**Table 1:** Teacher school and position.

Teacher	School (location)	Grade	Position
A	1 (City Center)	K	Classroom
B	2 (City Center)	PreK-5	Librarian
C	2 (City Center)	K	Classroom
D	2 (City Center)	2,3	Classroom
E	3 (Green schoolyard)	PreK	Classroom
F	1 (City Center)	PreK	Classroom
G	4 (Green schoolyard)	4,5	Classroom
H	2 (City Center)	K	Aide
I	3 (Green schoolyard)	3	Classroom
J	2 (City Center)	5	Classroom
K	2 (City Center)	5	Classroom
L	1 (City Center)	K	Classroom
M	2 (City Center)	3,4	Classroom
N	2 (City Center)	PreK,K	Classroom
O	1 (City Center)	2,3	ESL
Q	5 (Green schoolyard)	1	Classroom
R	3 (Green schoolyard)	PreK	Classroom

### Instrumentation

Data were collected through two different mixed-method Qualtrics surveys. Implementation surveys, distributed on the 15 randomly selected days, asked teachers to report if they took their class outside that day, for how long (minutes), the location (categorical responses; e.g., playground, outdoor classroom, garden, natural area) and activities (categorical responses; e.g., free play or free choice, instruction focused on a nature topic, etc.), as well as the reasons they went outside and the barriers that prevented them from going outside (open-ended qualitative responses). The *post-survey* asked teachers to reflect on what facilitated (or limited) their ability to integrate outdoor time into their classroom activities.

### Procedure

Preparation workshop: participants attended a two-hour professional development workshop conducted by the project team at the start of the project. The workshop covered the benefits of outdoor time, discussion of the barriers participants faced in incorporating outdoor time for their classes, and strategies for increasing outdoor time during school hours using a Toolkit (61) developed earlier in this project. Participants developed plans for the strategies they would use to incorporate outdoor time for their classes in the following weeks.

Implementation: for 8 weeks following the workshop, participants worked independently to incorporate outdoor time into their classes, in the manner that worked for each class. The project team did not prescribe any particular outdoor activities or suggest any amount of time to spend outside; participants were asked to incorporate outdoor time when and how it worked for them.

Implementation supports and resources: during the project participants were provided with access to the strategy Toolkit, email consultations with the project team, approval for the project from school principals, and $300 grants to support implementation. Participants were asked to make a plan for how they would use the funding during the workshop but were not required to stick to their predicted plans. Concurrently, outdoor gear (rain gear, boots) was provided to all elementary students through a separate district project.

Data collection: All data were collected through online surveys (Qualtrics XM). Implementation surveys were sent to participants at the end of the school day on 15 randomly selected days across the 8 weeks of implementation. The survey days were the same for all participants and identified by the research team before implementation so their selection would not be influenced by conditions week to week; participants were not informed which days would be surveyed. Participants were sent a reminder email if they had not completed the survey by the end of the next day. Weather conditions at noon on each of the survey days were collected from weather.gov by the research team. The post-survey was sent to participants 2 weeks after the 8 weeks of implementation.

### Analytical approaches

Implementation survey data: Descriptive statistics of quantitative (days and time outside) and categorical (activity, location) data were calculated in Excel. The number of days outside, number of days doing various activities, and number of days using each type of location were counted for each teacher and summed for all teachers. Qualitative data (motivations/reasons to go outside, limitations) were coded for themes using both pre-existing and emergent themes to identify common and unexpected factors. Thematic analysis was used to examine the factors that encouraged or prevented outdoor time across teachers. The number of days where each motivation category was mentioned by a teacher was counted for each teacher, and cumulatively for the cohort. The number of days each limitation theme was mentioned as a barrier by an individual teacher was also counted.

Post-survey data: Qualitative data on facilitators were coded for themes based on responses. The themes were then used as categories to identify factors that facilitated outdoor time across teachers. The number of teachers who mentioned each category was counted.

## Results

The 17 study participants completed 249 of 255 implementation surveys (98% response rate), and all completed the post-survey (100% response rate). Data indicated that across the study period, teachers were able to take their students outside, but the amount of time, activities, locations, motivations, barriers, and facilitators varied across participants and days. These are elucidated below.

### Days and time outside

Participants took their classes outdoors on 78% of opportunities (198 occurrences of 249 implementation surveys; Table 2). Outdoor time ranged from 6–15 outdoor days per class (out of 15 possible days), and 0–285 min per day (average of 104 min on days with time outside). A few teachers (e.g., C, Q) reported relatively consistent minutes outside across days, but for most teachers the minutes outside varied on different days. Younger grades generally reported both more days where they went outside and more minutes outside on those days than the higher grades. On average, kindergarten classes (*n* = 4) went outside on 12.5 days for 145 min/day, while 5^th^ grade classes (*n* = 3) went outside on 6.5 days for 34 min/day.

**Table 2:** Days and time outside by teacher.

			Teacher (grade)																
Date	Weather	Temp (F)	A	B	C	D	E	F	G	H	I	J	K	L	M	N	O	Q	R
			(K)	(PK-5)	(K)	(2,3)	(PK)	(PK)	(4,5)	(K)	(3)	(5)	(5)	(K)	(3,4)	(PK,K)	(2,3)	(1)	(PK)
20-Apr	Overcast	44	120	15	180	20	180	50	20	180	20	d	b	45	30	240	25	240	75
21-Apr	Heavy Snow	31	120	a	180	25	a, f	a	a,f	180	10	20	f	45	20	240	a	200	a
26-Apr	Partly Cloudy	39	120	40	180	50	120	50	a	180	45	45	30	50	120	255	120	240	120
30-Apr	Overcast	49	b^1^	40	180	30	120	40	30	210	15	f	f,g	75	60	135	120	240	90
5-May	Light Rain	49	*	40	180	50	120	c	20	210	a,d	d	f	20	20	180	75	240	120
6-May	Mostly Cloudy	44	120	30	180	45	120	50	30	210	40	25	f	45	35	270	80	240	45
7-May	Overcast	51	b	30	180	c	240	60	30	240	25	75	60	210	35	240	60	*	20
10-May	Overcast	49	120	d	180	60	f	50	25	210	30	d	25	100	40	c	60	210	65
11-May	Overcast	48	120	*	180	25	190	30	f	c	d,e	25	30	90	40	270	65	240	75
13-May	Partly Cloudy	60	120	35	180	25	240	45	30	c	30	30	30	45	40	285	60	240	120
19-May	Fair	77	120	40	c	60	210	*	c	c	50	20	f	60	65	*	60	180	45
21-May	Fair	84	b	40	180	50	180	c	50	c	c	g	f,a	240	c	210	90	240	40
25-May	A few clouds	63	60	35	180	25	200	60	c	c	20	d	f	60	75	275	d	240	180
2-Jun	Light Rain	63	120	40	180	45	140	60	30	210	a	a	30	60	50	270	90	120	d
3-Jun	Overcast	70	120	40	180	50	140	50	f	240	c	d	f	50	20	240	*	210	30
	Total days outside		11	12	14	14	13	11	9	10	10	7	6	15	14	13	12	14	13
	Avg min when outside		115	35	180	40	169	50	29	207	29	34	34	80	46	239	75	220	79

*Did not complete survey.

1 Barrier that prevented outdoor time: (a) weather, (b) distance learning day, (c) teacher absent, (d) curriculum need (tech, space, materials), (e) insufficient time, (f) schedule conflict, (g) not enough staff.

### Barriers that limited outdoor time

There were 51 instances where a teacher did not take their class outside on a surveyed day. Barriers that prevented classes from going outside on a particular day included teacher absence (15), schedule conflict (14), weather (11), curriculum needs (10), distance learning logistics (4), insufficient staff (2), and time pressure (1) (Table 2). Looking across teacher responses, most teachers who reported more than 1 day where they did not go outside reported more than one type of barrier. The three teachers that reported the fewest days outside all taught 5^th^ grade (Teachers G, J, K). For these three teachers, the most common barrier was schedule conflict (12 days), followed by curriculum need (5), weather (4), teacher absence (2) insufficient staff (2), and distance learning (1).

### Activities and locations

Teachers reported where they took their classes (location) and what type of activities they did on the days they went outside. Total locations and activities for each teacher are shown in Table 3. Many teachers reported more than one activity and location on a single day, indicating multiple or overlapping outdoor experiences. Free play (138) was the most common activity, followed by intentional engagement with nature (57), instruction focused on nature (44), instruction but not focused on nature (40), other activities such as lunch outside or special events (33), emotional or energy breaks (24), transit (11), and instruction specifically focused on sustainability (4). Human made spaces (playground, blacktop) were the most commonly selected location (138 instances), but natural spaces [school garden (89); outdoor classroom (81); other natural space (65)] were used more frequently when counted collectively (235 total instances).

**Table 3:** Locations and activities by teacher.

Location	Total^1^	Teacher																
		A	B	C	D	E	F	G	H	I	J	K	L	M	N	O	Q	R
Human-Made Space (playground, blacktop)	138	10	4	13	9	13	10	0	2	3	2	3	15	13	13	10	6	12
Natural Space (combined)^2^	235	21	10	27	7	18	10	1	18	12	4	3	19	6	31	6	30	12
School Garden	89	7	6	13	2	7	8	0	9	3	2	1	9	1	13	2	3	3
Outdoor Classroom	81	9	4	13	0	1	1	0	7	0	1	0	7	5	13	1	14	5
Other Natural space	65	5	0	1	5	10	1	1	2	9	1	2	3	0	5	3	13	4
Other	18	1	0	0	0	0	0	9	1	0	2	1	1	0	0	1	0	2
Activity																		
Free play or free choice time	138	11	0	14	6	12	10	6	10	4	2	1	14	13	13	3	9	10
Intentional engagement with nature	57	4	3	3	1	1	9	6	0	1	1	2	9	0	5	5	1	6
Instruction focused on nature topic (e.g., bugs, seasons)	44	11	2	0	0	7	0	1	0	4	3	3	5	0	0	2	3	3
Instruction not focused on nature topic (e.g., math)	40	0	2	0	0	3	0	0	0	0	2	1	5	1	8	1	13	4
Other (e.g., lunch, assembly)	33	0	5	0	0	0	0	0	2	1	0	2	2	3	1	2	13	2
Guided emotional or energy break	24	0	0	13	1	1	1	0	1	0	1	0	0	0	0	0	0	6
Transit (e.g., Walked back from music class outside)	11	0	0	3	0	5	0	0	0	0	0	0	2	0	0	0	0	1
Instruction focused on sustainability	4	0	0	0	0	1	0	0	0	0	0	1	1	0	0	0	1	0

1 Total refers to the number of individual daily surveys out of 249 that listed this location or activity.

2 Combined natural spaces may exceed 15 if more than one natural space was used on a survey day.

Most teachers utilized a range of locations. Some teachers used multiple locations fairly equally while other teachers preferred one or two locations and occasionally utilized others. For activities, nine teachers selected free play most frequently, three selected free play and another activity most often and equally, and only five selected a different activity more than free play. While all teachers did indicate conducting more than one type of activity outside over the 8 weeks, it seemed that most conducted a small number of activities frequently and only occasionally included others. Free play was the most frequent overall and identified as a frequent activity by the most teachers, but the other frequently-selected activities varied across teachers.

### Motivations

Teachers described the reasons they decided to take their classes outside that day on each of the survey days. Ten themes were identified in their responses (Table 4). Across the 249 implementation surveys and 17 teachers, the health benefits of time outside was mentioned the most often (68 individual surveys) and by the most teachers (14), followed by having time in the schedule on that day (58 surveys, 10 teachers), wanting to get out in nice weather (49 surveys, 12 teachers), doing an activity specifically to incorporate time in nature (41 surveys, 13 teachers), doing a planned content activity (e.g., reading, science) outside (32 surveys, 12 teachers), gardening projects (18 surveys, 7 teachers), a class reward for hard work (10 surveys, 4 teachers), improving the group dynamic (9 surveys, 5 teachers), the fact that the teacher wanted to (2 surveys, 2 teachers), and instruction that worked better outside (1 survey, 1 teacher). All but one teacher indicated more than one reason for taking students outside across survey days, and many indicated more than one reason on any given day.

**Table 4:** Teachers’ motivations for outdoor time across implementation period.

Reasons	Total times^1^	Total teachers^2^	Teacher																
			A	B	C	D	E	F	G	H	I	J	K	L	M	N	O	Q	R
Total per teacher			3	4	6	3	6	7	4	2	4	5	1	6	8	6	6	4	5
Health	68	14	1	0	6	9	8	7	2	1	2	0	0	3	11	6	5	1	6
Time	58	10	0	0	7	5	0	1	0	10	1	0	0	9	3	7	1	14	0
Weather	49	12	0	2	7	0	9	2	4	0	0	1	0	2	4	3	6	2	7
Nature Incorporation	41	13	2	2	2	4	2	4	5	0	5	3	0	1	3	0	2	0	6
Content Activity	32	12	0	6	3	0	1	1	0	0	2	2	6	3	1	2	4	0	1
Gardening	18	7	1	4	0	0	3	3	0	0	0	2	0	4	1	0	0	0	0
Reward	10	4	0	0	0	0	4	0	0	0	0	1	0	0	4	0	0	0	1
Group Dynamic	9	5	0	0	1	0	0	4	0	0	0	0	0	0	2	1	1	0	0
Teacher Interest	2	2	0	0	0	0	0	0	1	0	0	0	0	0	0	0	0	1	0
Instructional Need	1	1	0	0	0	0	0	0	0	0	0	0	0	0	0	1	0	0	0

1 Total times refers to the number of individual daily surveys out of 249 that listed this reason.

2 Total teachers refers to the number of individual teachers who listed this reason at least once.

### Facilitators

In the post-survey, teachers reflected on the factors that helped them incorporate outdoor time in their teaching. Three themes, with 10 categories of supports, were identified in their responses (Table 5). Seven teachers mentioned just one support in their reflection, eight mentioned two or three, one mentioned six, and one did not answer the question. *Encouragement*, in the form of supportive colleagues or nudges from the project, appeared to facilitate outdoor time. Having supportive colleagues was mentioned most often, with teachers stating that having a supportive principal (6) or co-teacher (2) facilitated outdoor time. Five teachers mentioned that knowing we might ask them on the implementation survey served as a reminder and encouragement to have something “worthwhile” to report. Second, teachers identified *structural or logistical* supports. Teachers mentioned having appropriate weather gear for all students (5), sufficient and appropriate space for the outdoor activity (3), flexible schedules (3), funding for supplies (2), sufficient staff (2), and connections to the curriculum (1). Third, *intentionally planning* to include outdoor time was mentioned by three teachers. Of note, this is the only facilitator that teachers independently controlled.

**Table 5:** Factors that Facilitated Outdoor Time by Teacher.

Facilitator	Total^1^	Teacher																
		A	B	C	D	E	F	G	H	I	J	K	L	M	N	O	Q	R
Total per teacher		2	1	3	2	3	2	1	0	1	1	1	1	6	2	1	3	2
Encouragement	13																	
Supportive admin	6	1		1			1							1	1			1
Supportive co-teacher	2												1				1	
Reminders	5		1					1		1				1				1
Structural and logistical	16																	
Weather gear	5			1		1								1	1	1		
Appropriate space	3	1				1								1				
Flexible schedule	3										1	1					1	
Funding for supplies	2			1		1												
Sufficient staff	2						1							1				
Curriculum	1				1													
Intentional planning	3																	
Planning to go out	3				1									1			1	

1 Total column indicates the total number of teachers that mentioned each facilitator.

## Discussion

The purpose of this study was to understand whether and how elementary teachers could consistently incorporate outdoor time in their classes, to guide our efforts to increase the time elementary students spend in nature during school time. We were particularly interested in understanding how time in nature could be frequently and consistently integrated into the elementary school day, rather than as an occasional or intermittent component, to better support students’ mental wellbeing. The findings from this study suggest that outdoor time can be frequently and consistently incorporated into elementary class schedules, but the duration, opportunities, supports, and challenges are not uniform either across classes or for individual teachers across days. Additionally, findings suggest that focusing on free play, highlighting the health benefits, and using a flexible and systems-based approach may help increase implementation. These points are outlined below.

### Consistent outdoor time was possible, but implementation varied

Teachers in this study were able to incorporate consistent outdoor time into their class routine over the 8 weeks of the study, but the activities, amount of time, and barriers varied across teachers and days. Our request to teachers during the 8 weeks of the project was intentionally open-ended, encouraging teachers to do what worked best for their classes, to identify a range of possible activities. Across the study period, all 17 teachers were able to take their students outside for at least 10 min (a minimum “dose” needed for mental health benefits) (16), though the number of days they were able to do so varied across teachers. Extrapolating from the surveyed days to a full week suggests that some would reach the 120–300 min per week threshold (37), but others would not. Collectively, teachers utilized all the locations and activities asked about in the surveys. This aligns with locations (2, 44, 46) and activities (2, 3, 45–47) identified in previous literature, and adds some outdoor experiences that were not related to a specific instructional or physical activity goal (e.g., transit, outdoor lunch). Further, the data showed variation in the activities and locations teachers used. None of the locations or activities were utilized by all 17 teachers during the study period (each individual location/activity was not used by at least one teacher). All 17 teachers used a variety of activities and locations across the study days, and the activities that teachers chose to incorporate shifted from one day to the next. This suggests that different activities were feasible at different times, even within the same school and class. Utilizing multiple types of activities (e.g., instruction, free play, physical activity, social–emotional breaks) in multiple locations as they are possible for a class on a given day could increase the feasibility of frequent and consistent outdoor time in the elementary school day. These data add to previous work by documenting teachers’ use of multiple activities and locations across instructional topics or outcome goals to give a more comprehensive picture of how outdoor time can be integrated into the school day and the amount of time teachers are able to incorporate.

Similarly, the barriers that prevented outdoor time varied between teachers and across days. Some teachers were consistently able to take their students outside, while others experienced more barriers that prevented outdoor time. The barriers that prevented teachers in this study from taking their classes outside align with barriers identified through previous research (1, 6), as does the fact that the barriers shifted across classes and days (48). Participants in this study were a coalition of the willing, but, as in previous studies (48, 50, 55), interest was not always sufficient to overcome the dynamic barriers. This was particularly true for older grades: the majority of the schedule conflict (12 of 14) and half the curriculum conflict (5 of 10) barriers reported across teachers were for the three 5^th^ grade classes. This aligns with the lower prevalence of outdoor learning in upper grades in the Scandinavian countries (34, 35), where the cultural and institutional support for udeskole should theoretically reduce barriers. Further research into barriers that arise in upper grades could help support continued implementation. Additionally, as participants in this study were volunteer early adopters, and most were teaching younger elementary grades, research with a broader range of teachers would help identify factors affecting implementation among teachers who were not as eager or faced additional barriers in upper grades. Barriers that shift across classes and days suggest that flexible strategies will be required to increase consistent outdoor time.

### Free play may be easier to implement consistently than content

Teachers in this study were most frequently able to incorporate outdoor time as free play, and on human-made play locations (e.g., playground); connecting outdoor time to curriculum was not as common. While this was certainly influenced by the fact that specific curricular connections were not required or provided in this project, it also points to the challenges teachers may have making these connections when it is not built in to the curriculum. Teachers in this study were able to connect curricular pieces to outdoor experiences at times, it just happened less often than outdoor breaks or free play. This aligns with previous research that identified challenges with connecting outdoor learning to the curriculum and implementing content-specific outdoor instruction (6, 51, 53–55). Teachers who are independently interested in incorporating outdoor time into their class day without the structure of a guided curriculum project may find free play to be an easier approach. Although this may not support deeper learning on specific content, unstructured free play in settings where there is nature present (e.g., playground surrounded by trees) could support cognitive development and stress reduction outcomes (7–9). Previous research on attention benefits suggests that outdoor free play may enhance focus during subsequent indoor instruction (10), thereby enhancing content learning. Encouraging and supporting outdoor free play at times other than recess may increase access to the cognitive and mental wellbeing benefits. Additionally, as opportunities for free play typically decrease in older grades, this suggests that older grades may need a more structured and intentional approach to support implementation, and one that considers the interaction of factors across the school system (48, 50). Tying outdoor time to curriculum is a great structured option when it works, but also a barrier to implementation when it does not. Further research focused on the particular factors that make curricular connections possible or challenging could identify ways to make outdoor content learning more feasible for more teachers. Research into structured alternatives, such as scheduling nature breaks into the day, or a nature-based “special” similar to music or art could also potentially support implementation in settings where curricular connections or independent action by individual teachers are not possible.

### Focusing on health connections may help increase outdoor time

Highlighting the health benefits of time in nature could help increase educators’ motivation to take students outside. Teachers in this study mentioned multiple factors that motivated them to take students outside that connect to previous work. The desire to incorporate nature aligns with teacher interest and environmental attitudes identified as motivators in previous work (50, 52, 57). Teachers’ statements that nice weather and having time in the schedule motivated them to take students outside identifies potential positive impact from factors that were also identified as barriers, in this study and previous work (49, 51, 53). Unlike previous work, particularly related to outdoor environmental or science learning (3, 57), teachers did not mention curriculum connections as a frequent motivator. Most of the motivations in this study, including the four mentioned most often (health, time, weather, nature incorporation) were not related to specific content learning. A majority of teachers (12) did mention that doing a specific content activity motivated them to take students outside on at least one surveyed day, but health benefits, easy logistics, and the desire to be outside in nature were the main drivers.

Interestingly, the most common motivation in this study—wanting to take advantage of the health benefits—is not as prominent in other work on teachers’ reasons for using outdoor learning. Health benefits appeared to motivate teachers in this study even when there was not a curriculum-related reason to take students outside. Further, the data on the health benefits from time in nature is substantial (4, 5, 7–9), connects to many types of outdoor experiences, and is relevant across all grades and subjects. These findings suggest that individuals seeking to plan interventions or increase outdoor time may find it useful to highlight the health benefits in framing their projects.

### Flexibility and a systems view may increase opportunities

Finally, the findings suggest that taking a flexible approach to implementation that accounts for dynamic interactions in a complex system may increase opportunities for students to spend time outside. A common theme across the findings was the variation in what teachers were able to do, what motivated them, and what helped or hindered implementation, even for one teacher on different days. This variation speaks to the dynamic systems in schools, and the many shifting factors that influence what teachers are able to do (48, 50). Multiple options would allow teachers to incorporate the one that fit for their classes in the moment, and adapt as the moment shifted. Flexibility in implementation may increase opportunities and lead to more successful implementation of outdoor time than a one-size-fits-all directed approach.

Additionally, teachers in this project suggested an array of factors that facilitated or limited outdoor time. The majority of these factors were beyond the control of the individual teacher, with several (school schedule, resources, appropriate outdoor spaces, sufficient staff, administrator support) more likely to be coordinated at the school or district level. This aligns with previous research that identified supportive administrators, resources, staffing, appropriate spaces, and schedules as factors that affected opportunities to take students outside (1, 49–51). The fact that these factors are outside the control of individual teachers points to the importance of coordinated support for outdoor learning. The facilitator mentioned most often—encouragement in the form of a supportive colleague or the psychological “nudge” to have something to report on the project survey— aligns with the interpersonal level of a multi-level understanding of the school system (50) and presents a relatively simple opportunity to encourage implementation of nature exposure. Coordinating supportive colleagues or accountability partners across a school could leverage this interpersonal facilitator to increase prevalence of nature exposure. This echoes previous work (50) and the findings from Phase 1 of this project (48), which suggested that taking a systems approach to address shifting barriers could help increase consistent outdoor time.

### Limitations

This was a small study in a single context. The results can inform practice for other schools, but are not generalizable across all settings. In particular, since even the schools in the city center in this location had some greenspace in the schoolyard, the sample does not capture the challenges in accessing greenspace that may be present in more urban contexts. Repeating the study with a larger sample, with participants that were both eager and reticent about taking students outdoors, across a longer time span, and in different contexts could add reliability and detail to the findings. Student level data (socioeconomic status or demographics) and teacher background experience were beyond the scope of this project but would support comparison across locations in future studies. Additionally, data collection occurred when schools were dealing with the COVID-19 pandemic. This may have influenced motivations, considerations, and implementation.

## Conclusion

This study builds on understanding of how teachers may be able to frequently and consistently implement outdoor time during the elementary school day. While not broadly generalizable, the implementation data provide a detailed day-to-day look at how teachers in this school district were able to incorporate outdoor time, how long students spent outside, teachers’ motivations, and the factors that facilitated and limited opportunities. Data showed variation across teachers and days. Teachers were most consistently able to implement additional outdoor free play, but most utilized a range of activities across days. Likewise, the factors that limited outdoor time shifted for individual teachers across days. Encouraging multiple types of outdoor activities, and supporting flexible implementation, may be more effective at increasing consistent outdoor time than focusing on any particular structure or activity. Highlighting the health benefits of outdoor time and examining the dynamic system of facilitators and barriers may help encourage frequent and consistent use of outdoor time to support health and development across classes and grades.

## Data Availability

The raw data supporting the conclusions of this article will be made available by the authors, without undue reservation.
